# Designing Tangibles to Support Emotion Logging for Older Adults: Development and Usability Study

**DOI:** 10.2196/34606

**Published:** 2022-04-27

**Authors:** Daniel Gooch, Vikram Mehta, Avelie Stuart, Dmitri Katz, Mohamed Bennasar, Mark Levine, Arosha Bandara, Bashar Nuseibeh, Amel Bennaceur, Blaine Price

**Affiliations:** 1 School of Computing and Communications The Open University Milton Keynes United Kingdom; 2 Department of Psychology University of Exeter Exeter United Kingdom; 3 Department of Psychology Lancaster University Lancaster United Kingdom; 4 Lero, the Science Foundation Ireland Research Centre for Software University of Limerick Limerick Ireland

**Keywords:** older adults, health, emotion, affect, well-being, tangible interaction, TUI

## Abstract

**Background:**

The global population is aging, leading to shifts in health care needs. In addition to developing technology to support physical health, there is an increasing recognition of the need to consider how technology can support emotional health. This raises the question of how to design devices that older adults can interact with to log their emotions.

**Objective:**

We designed and developed 2 novel tangible devices, inspired by existing paper-based scales of emotions. The findings from a field trial of these devices with older adults are reported.

**Methods:**

Using interviews, field deployment, and fixed logging tasks, we assessed the developed devices.

**Results:**

Our results demonstrate that the tangible devices provided data comparable with standardized psychological scales of emotion. The participants developed their own patterns of use around the devices, and their experience of using the devices uncovered a variety of design considerations. We discuss the difficulty of customizing devices for specific user needs while logging data comparable to psychological scales of emotion. We also highlight the value of reflecting on sparse emotional data.

**Conclusions:**

Our work demonstrates the potential for tangible emotional logging devices. It also supports further research on whether such devices can support the emotional health of older adults by encouraging reflection of their emotional state.

## Introduction

### Background Context

The United Nations predicts that the global population aged 60 years and older will increase from 962 million in 2017 to 2.1 billion in 2050 and 3.1 billion in 2100, making this the fastest growing age group [1]. These demographic changes will significantly impact how we think about supporting the health and well-being of the population. Older people can face long-term disabilities and chronic conditions as well as mental health difficulties [2]. For example, Age UK has noted that the number of over-50s experiencing loneliness is set to reach 2 million by 2025/6. This compares to around 1.4 million in 2016/7—a 49% increase in 10 years. For the purposes of this work, the term “older adults” is used to refer to anyone over the age of 50 years based on the recommendations of Age UK (the main charity working with older adults in the United Kingdom).

This increase in the older population will drive an increase in the need for carers and the costs of health care [3]. This has led to significant amounts of research into how to enable people to age in place; “the desire and tendency of older persons to stay in their current dwelling units for as long as possible” [4]. Compared to other forms of care, aging in place is more cost-effective and preferred by many older adults [5]. This is because it can enhance many quality of life factors (eg, identity, autonomy, belonging, privacy, independence, social connections) [6,7].

There have been promising developments in the design of technology to support the physical health of an aging population [8-10]. However, there is increasing recognition of the link between well-being and “successfully” aging, which makes it important to improve the psychological well-being of older adults [11]. This necessitates mechanisms for the detection or logging of the older adult’s emotional state to either ensure that the older adult is happy or provide appropriate support when in emotional turmoil [12-14].

Although a wide variety of digital technologies have been developed for the monitoring of emotions [15-23], there is little work that explores such interfaces specifically for older adults [24]. In a review of apps for successful aging, no apps for monitoring emotions were identified [25]. Given that older adults have distinct cognitive, physical, and technical skills, alongside distinct emotional needs, it is necessary to consider the design of a system for recording the emotional state of older adults at home [12,26].

Many researchers argue that tangible user interfaces (TUIs) are ideal for use in domestic settings by older adults owing to both their acceptability in domestic settings and the comparatively quick learning curve [27-29]. TUIs allow the user to provide input to a digital system by manipulating physical objects (eg, moving them around or stretching and squeezing them). Similarly, output from the TUI interaction could be shown to the user through the manipulation of a physical object. TUIs have also been found to increase engagement with logging emotions, suggesting that this form factor could promote ongoing use [17]. A broad review of the TUI literature for supporting social interactions among older adults highlights that most papers conclude that TUIs are highly usable for older adults [30].

In previous laboratory-based work, we have demonstrated that nonfunctional prototypes of tangible devices allow older adults to log emotions and collect data comparable to validated psychological scales of emotion [31]. We build on this work by developing 2 of these nonfunctional prototype designs into tangible devices that can digitally record the logged emotions. Our field study with adults aged 51-85 years demonstrates the validity of logged data against existing scales of emotion, showing that tangible devices can provide data comparable to standard psychological scales in a home setting. We explored our participants’ experience of using the devices over a 6-week period. This provided an understanding of how users can appropriate the use of the devices as well as how key design characteristics are viewed. Our results highlight the potential of in-home tangible devices for recording the emotions of older adults and for supporting their emotional health through encouraging reflection of their emotional state.

### Background Literature

By exploring previous approaches to logging emotion, we can identify key design properties that should be embedded in the design of tangible devices for logging emotions. Through exploring the literature on self-report scales of emotion, interfaces of logging emotion, and TUIs for logging emotion, we identify key design decisions and reflect on them when outlining the development of our TUI devices in the section “Designing tangible devices for logging emotions.”

It is important from the outset to distinguish between emotion and mood. Although both refer to phenomenological states, they differ in 2 key dimensions [32,33]. The first is time; emotions tend to be short-lived, whereas moods are more enduring. The second difference is that emotions are object-driven (ie, they relate to a specific object or experience), while moods are more general. The concepts are related; a person’s mood biases the emotions they experience and a person’s emotions contribute to the mood they are in. Throughout this paper, the term “mood” is used only when it is the term used by other researchers in their work. The terms “emotion” and “affect” are used interchangeably as is common practice [33].

Across all fields interested in emotional experience, there are 3 main approaches to detecting and measuring how people feel: physiological, behavioral cues, and self-report. This research is focused on self-reported measures of emotion. Although self-report measures have shortcomings, they provide the user with a level of control over the disclosure of their emotional state. This is important for older adults in having an active role in their health care needs [34,35]. Self-reporting emotions also has other benefits. From a well-being perspective, there is a rich literature on the benefits to an individual of emotional reflection and recording, which is commonly used as a therapeutic technique [36]. Studies are starting to show how technologically-mediated reflection and recording can improve well-being [37] and promote behavior change [38]. From a methodological perspective, a recent review of ecological momentary assessment of mood highlights the importance of self-reporting due to ecological validity and agency [39].

### Self-report Scales of Emotion

There are many different measures and scales focused on emotion in the psychology literature. Desmet et al [15] provide an excellent review of this literature. These measures predominantly coalesce around 2 concepts: valence (pleasure) and arousal (strength of feeling). Dominance is a third concept that is also sometimes used [40]. Proponents argue that these 3 dimensions can account for significant variances in people’s emotional experiences and collectively correspond to affect.

**Figure 1:**
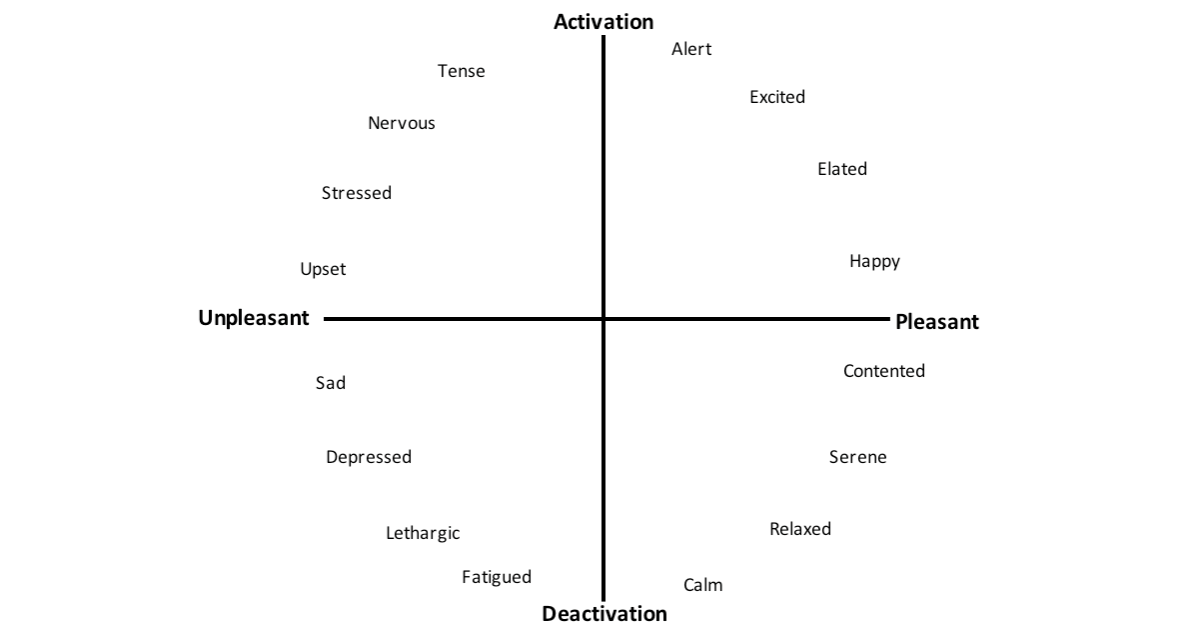
A schematic for the 2D structure of emotion from [41]. The valence scale runs left-to-right and the arousal scale runs top-to-bottom.

Russell’s 2D approach to conceptualizing emotion is one of the most popular measures of emotion [41,42]. He models emotion as a spatial distribution across 2 scales (valence and arousal) (see Figure 1). This approach argues that a spatial model provides a conceptual structure for related emotive concepts in such a way that allows the self-reporting of emotions [41]. A related approach uses emotive words to distinguish between related emotive states. One of the first commonly used robust measures that took this approach was the Semantic Differential Scale, consisting of a set of 18 bipolar adjective pairs [43]. Each pair is then rated along a 9-point scale. Although heavily used, the measure is extremely cumbersome to use, requiring 18 different measurement ratings for each stimulus. It also relies on an individual’s English reading skills.

A variety of pictorial scales have also been developed. The Self-Assessment Manikin (SAM) is a classic example, made up of 3 pictorial scales: for affect (or valence), the pictures range “from a smiling, happy figure to a frowning, unhappy figure;” for arousal, the pictures range “from an excited, wide-eyed figure to a relaxed, sleepy figure;” and for dominance, the change is in the size of the figure, “a large figure indicates maximum control in the situation” [40]. Although SAM is straightforward to conceptualize, it is somewhat complicated to administer, particularly in terms of explaining the dominance dimension. Some have argued that the only intuitive scale is valence (positive vs negative facial expression) [15].

Alternative pictorial scales have aimed for even greater simplicity. For example, the “smileyometer” was developed as a single Likert-scale style set of emotive faces [44], while Desmet et al [15] generated 8 cartoon figures to represent key emotions. A questionnaire-based study with 191 participants suggests that their scale can provide robust and reliable assessments of individuals’ emotions.

All of these scales were designed to be completed on paper. Given that we are designing an interactive technology for the collection of emotional data, we now explore the literature on interfaces for collecting self-reported emotions.

### Interfaces for Logging Emotion

“A wide range of digital symptom monitoring tools exist, but there is a lack of evidence regarding their effectiveness in a health care context, particularly in the area of mental health” [45]. Much of the evidence that exists focuses on apps for people with mental health disorders (such as bipolar disorder) [46-51]. The findings from these studies highlight which design properties have led to beneficial results and thereby provide insights into the design properties to utilize in the development of tangible alternatives.

An 8-week randomized trial of a suite of 13 mental health apps by Zhang et al [49] identified 3 distinct user behaviors: learning, goal setting, and self-tracking. Most importantly for our interests, participants who engaged in self-tracking experienced reduced depression symptoms. This is significant, as it suggests that logging affect can lead to improved emotional well-being. Zhang et al [49] also found that “greater amounts of engagement did not necessarily lead to greater reductions in depression.” This is an important design principle as it highlights that the device does not necessarily need to repeatedly harass users to enter data; as long as they engage with the system, they will receive some form of benefit.

True Colours is a digital tool for monitoring mood disorders. Used by over 36,000 individuals, it has formed part of 21 unique research and clinical service settings in the United Kingdom [45]. In addition to providing additional evidence of the efficacy of the digital logging of affect, the authors also note that the technology provides many advantages over hard copy symptom monitoring diaries, including the ability to prompt for input and the ability to easily visualize changes over time [45].

Chandrashekar [50] has reviewed meta-studies of the use of apps for people with depression, anxiety, and schizophrenia. In addition to demonstrating that these apps can have clinical benefits for these conditions, they also established some characteristics of high-efficacy apps. Among other features, providing a simple user interface and minimal usage reminders were highlighted as helping provide benefits to users.

Beyond these apps developed to help people with mental health disorders, there are a variety of interfaces that draw on self-report constructs of emotion to support the logging of emotion based on pictorial scales [15] or Russell’s circumplex model [41,42]. None of these studies involved older adults, and the study focus was on exploring the developed design rather than the efficacy for users.

Fernández et al [52] developed a digital diary, specifically designed for older users. Users were encouraged to complete predefined questions about self-care and emotions answered on a tablet device. Fernández et al [52] focus on the usability elements of their design and field-tested the system with 10 participants aged over 60 years, who used the device for 5 days. Nine of the participants agreed that they would like to continue using the tool, and data collected from the study suggested that the simple act of logging was sufficient to prompt users to reflect about their day and how they were feeling.

Although the use of these interfaces has identified certain design properties as significant, they are not tangible devices. We now explore the sparse literature on TUIs for logging emotion to identify design properties specific to this interaction paradigm.

### TUI for Logging Emotion

A small number of tangible interfaces has been developed to log emotions. The EmoBall [53] used an LED matrix grid to display “faces” with positive (smiling) or negative (frowning) faces. When the ball is pressed, the display shows a face depicting a different emotion; when the ball is pressed twice, the displayed emotion is logged and the ball vibrates. While evaluated through focus groups with 16 people, the study investigated the usability of EmoBall for people with “low digital competences” rather than its efficacy as a mood logging device.

In a different context, the subtle stone was developed to allow students to privately share their affect with their teacher within a classroom setting [54]. A ribbed rubber ball, the subtle stone contained 6 LEDs, which could display 7 separate colors. Each student could develop their own color/emotion mapping, and an emotion is selected by repeatedly squeezing the ball until the color is shown. This was field-trialed with 15 UK school students (aged 12-13 years) throughout 9 hours of German language lessons, with students reporting that the device “supported reflection on emotional experience by giving them a way of thinking about their emotions.”

The Mood TUI was developed to make mood collection fun and engaging [17]. Designed as a cube with a different emoticon on each face, users select a mood by rotating the cube until the desired emoticon is facing upwards. Evaluated through discussion sessions with 32 participants, Sarzotti [17] concludes that there was interest in the design concept.

Jingar and Lindgren [55] took a design-oriented approach, co-designing TUIs to support the emotional health of older adults. Their interest was in how emotions could be communicated to a digital agent through tangible interactions. The variety of prototypes developed highlights the scope of the design space and the potential of TUIs to support older adults. Analyzing the data from their workshop, Jingar and Lindgren [55] argue that the nature of TUIs means that they may be “intuitive and natural to use, and intrinsic motivation may be promoted” [55].

Our previous work has highlighted the value of TUIs, particularly for those older adults who have arthritis or other musculoskeletal difficulties. Arthritis is a common condition, particularly in later life [56], and musculoskeletal difficulties can limit an individual’s ability to control a graphical user interface [57]. This makes tangible devices extremely suitable for use by older adults.

### Research Objectives

Although there is substantial literature on developing apps, interfaces, scales, and measures for logging emotion, few are explicitly designed for older adults ([15-23] focus primarily on younger adults). We are specifically interested in designing tools to support older adults to log emotions; therefore, we draw on this work for inspiration. Given that research highlights the potential benefits of designing TUIs for older adults, we specifically focus on designing and developing novel tangible devices. Taking inspiration from existing paper-based scales of emotions, we explore what design properties are valued by older adults in the context of monitoring their emotional state. From the literature in the background section (see Multimedia Appendix 1) [16,17,20,31,45,49-55], the key design considerations that appear to have a significant impact on participants’ use of the devices were to (1) minimize prompting, (2) ensure a clear mapping between the TUI interaction and the mood to be logged, (3) minimize fine grain movement, and (4) ensure that devices had a high-quality finish, suitable for use in a home location.

### Designing Tangible Devices for Logging Emotions

We build on our earlier work on mood logging [31] to explore (1) whether digital TUIs can log emotional data comparable to validated psychological scales of emotion and (2) whether such devices would engage older adult participants and what their view of particular design characteristics were after using the devices in a home context. Thus, our first design decision was to focus on TUIs and convert the validated nonfunctional prototype designs into digital devices.

#### Key Design Decisions

Stepping back from the intricacies of particular device designs, it is necessary to discuss one of the underlying psychological practices that supports the efficacy of logging data: reflection. Reflection is a key part of all logging behavior. Manual data collection can support the process of reflection in action [58]. In the context of logging emotion, it is well-established that taking the time to consider your emotional state has benefits in itself, particularly in terms of someone deciding to change behavior based on their reflection [34,35,59-62].

Our second design decision was to provide the device users with no access to their recorded data during typical use. Users would only be shown their collected data at the end of the field deployment and if they asked to see it (to promote the transparency of the research). This stands in contrast to many self-logging devices but allows us to explore any benefits of engagement with the data creation process, without confounding it with the benefits of reflecting on the historical data.

Our third design decision was to require minimal interaction [63,64], a design property that can help reduce the potential high burden of manual tracking. Given the perceived time burden of manual tracking [60], leading to high attrition rates [65], by minimizing the users’ interaction with the device, the potential time burden is also minimized.

#### Selecting the Emotion Scale

The background section highlighted the wide range of available emotion scales. Our previous exploration of nonfunctional prototypes using 3 distinct scales indicated that 2 of the scales should be developed further into digital devices. The prototype based on the emotive words from Russell’s circumplex were liked by users, given the simplicity of interaction and the speed of use. The prototype using the circumplex itself was liked by users, as it supported a more free-flowing process of reflection about their emotional state [31]. We decided to use these 2 scales of emotion.

**Figure 2:**
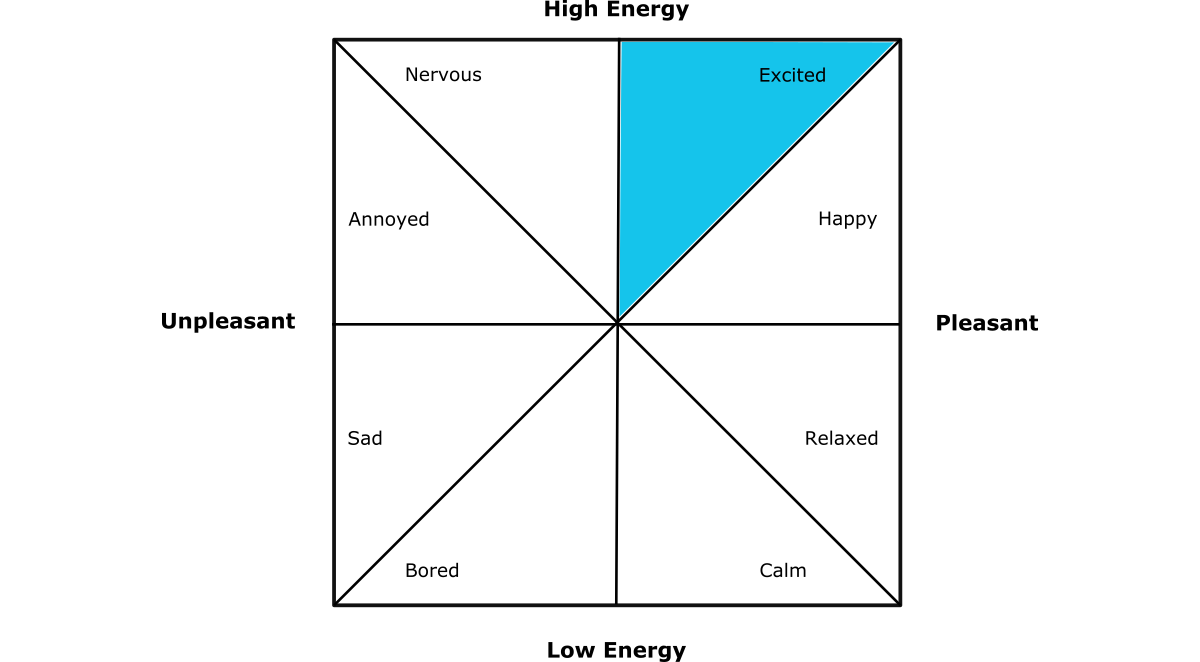
A representation of how the circumplex of affect [40] and the emotive words from [40] are both representations of the same scale.

Note that because these 2 scales represent the same conceptualization, analyzing the accuracy of logged data becomes easier. Figure 2 shows how the 2 scales can be considered to be somewhat equivalent. Taking the emotion of “excited,” the blue-highlighted octant can be taken to represent the emotion “excited” in the circumplex, and it is represented by the word “excited.”

We chose to focus on developing devices that can record 8 emotions: happy, excited, nervous, annoyed, sad, bored, calm, and relaxed. These 8 emotions provide wide coverage over the range of potential emotions and are a commonly used subset of representative emotions [15].

### Designing the Devices

Our previous work [31] focused on the development of nonfunctional prototypes of TUIs, which fulfilled the need to require minimal interactions [50,63,64]. As we have previously reported the design and development of these prototypes, here, we focus on the physical and electrical design of translating the nonfunctional paper prototypes into working digital TUIs. The resulting designs were named the Emotion Clock and the Emotion Board. These devices were developed by drawing on the design characteristics highlighted through the papers in the background section, in constant conversation with experts at Age UK to ensure that the resulting designs would be appropriate for use by older adults.

#### Emotion Clock

**Figure 3:**
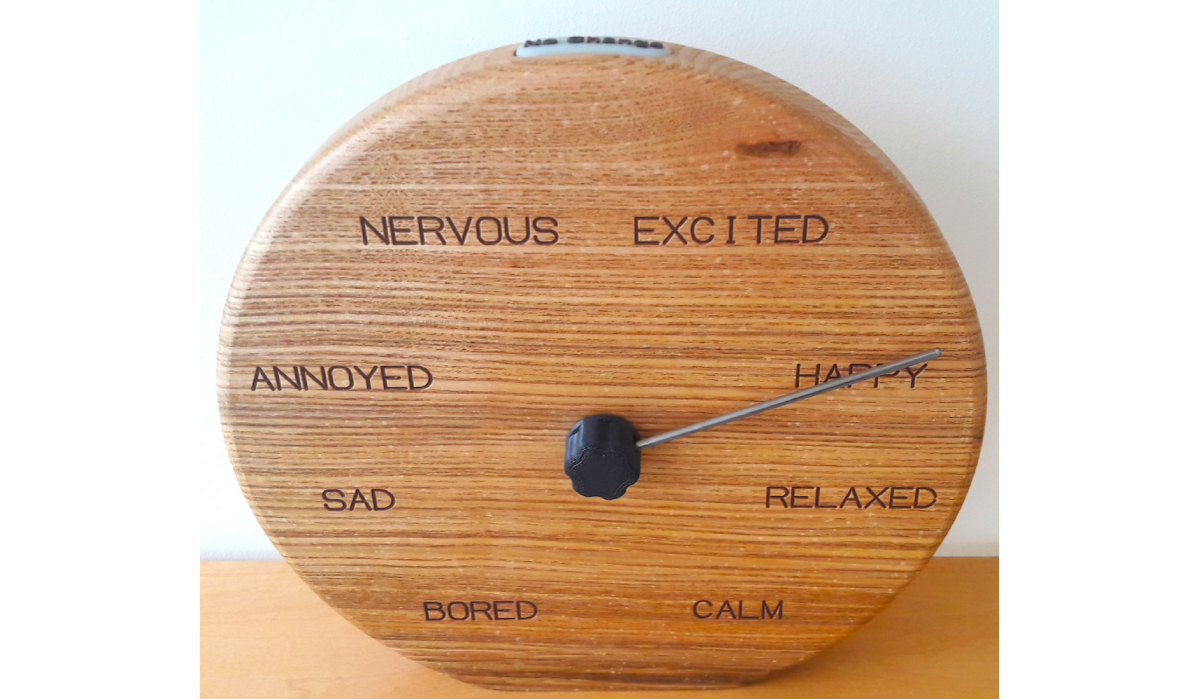
The Emotion Clock, using a subset of the emotive words in [40]. The emotion is set to Happy.

The Emotion Clock arranges 8 emotive words around a clockface in accordance with Russell’s valence/arousal circumplex [41,42] (see Figure 3). A user selects an emotion by rotating the clock hand to the word describing the emotion they want to convey. The words are engraved into a wooden clock face, with the electronics hidden in a recess behind the clock face. The Emotion Clock has a diameter of 26 cm. Users were not instructed on how to use the hand. Although the clock allows users to record on a continuous scale, leaving the hand between 2 words, for the purposes of analysis, the nearest word to the hand position is recorded.

#### Emotion Board

**Figure 4:**
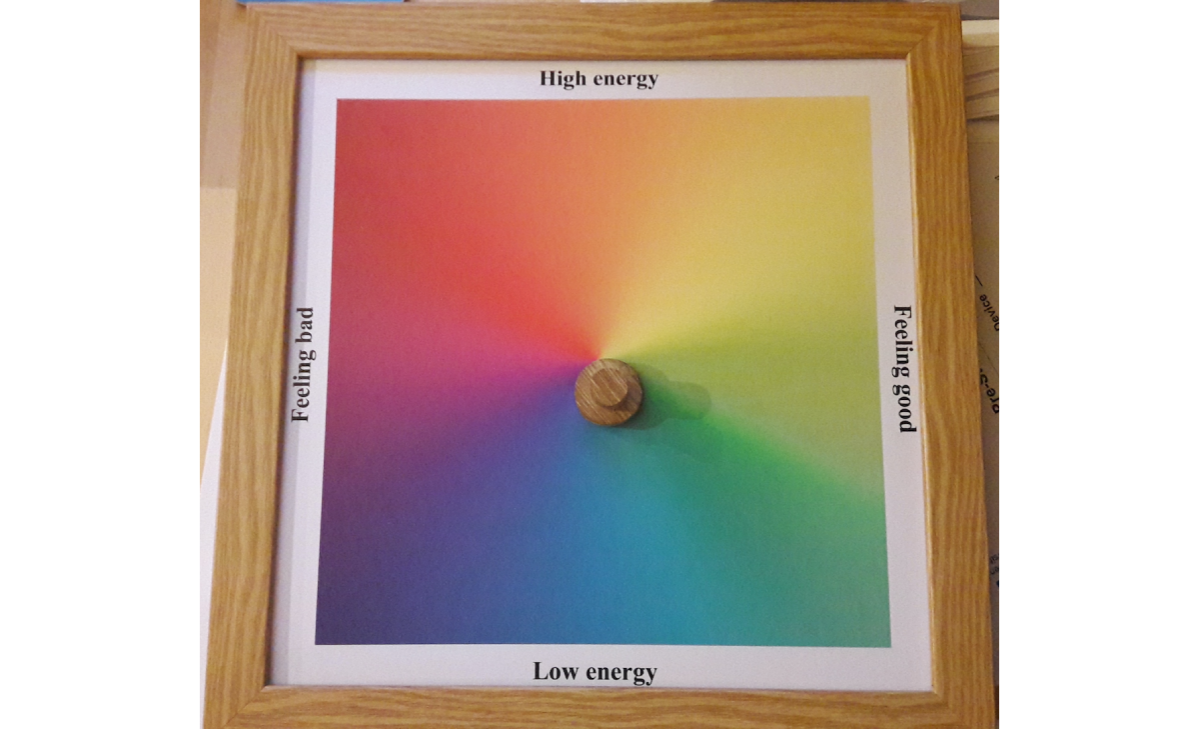
The Emotion Board, based on the Russell axes in [40] using the color scheme from [20]. The emotion is set to Calm.

The Emotion Board is a tangible representation of Russell’s axes [41,42], using the color scheme from Rivera-Pelayo et al [20] (see Figure 4). The axes are labelled High Energy to Low Energy (top to bottom) and Feeling Bad to Feeling Good (left to right). A user moves a magnet around to select a position on the axes and thus represent an emotive state. Framed in wood, there are 2 versions of the electronics behind the Emotion Board. The first version uses a custom piece of eTextiles, which is segmented to represent 16 sections of the axes (a high-arousal and low-arousal area for each of the 8 emotions). The second version uses an array of reed switches to achieve the same result but at a significantly lower cost. The board is approximately 26 square centimeters.

#### Logging Behavior

The 2 devices adopted the same approach for how the underlying electronics capture the logged emotion. Both devices were controlled by a custom circuit board that could receive the logged mood from the device and transmit the log to a Raspberry Pi over Wi-Fi using the Message Queuing Telemetry Transport protocol. The Raspberry Pi was connected to the participants’ router and could then send the log to our server using HTTPS.

The devices are powered using AA batteries rather than mains power. This allowed users to place the devices wherever they would like in their homes and improved the aesthetics of the devices by removing trailing wires. New batteries are sufficient to power the devices for at least 3 weeks. During the field trial, none of the participants’ devices ran out of power. One implication of this decision is that the electronics must be low powered so that users do not have to repeatedly replace the batteries. As such, the electronics are programmed to capture the recorded data in a targeted way. Each log is recorded on a central server rather than locally on the device. This allowed us to monitor whether a deployed device was working. It also meant that we could keep an accurate record of the logged data without having to worry about the device being damaged and losing locally stored data.

Both devices “woke up” every 5 minutes to check the position of the clock hand or magnet. If the position had not changed (indicating no new emotion input), the device went back to sleep and nothing was recorded. If the position had changed, the device sent the new emotion to our servers over Wi-Fi and recorded it locally (replacing the previously recorded emotion). The device would try to send the data to the servers up to 10 times before returning to sleep; if it had not successfully sent the emotion, it would attempt to send the locally recorded emotion the next time it woke up—this would continue until the batteries ran out.

Following advice from the literature, the devices prompt users to log their emotions regularly but infrequently to ensure sufficient reflection without placing an undue burden on users [45,49,50]. The devices beeped at noon and 6 PM for 5 seconds irrespective of how many inputs were given by the user for that date. The devices did not beep at any time outside this window. To encourage at least 1 logging action per day, between noon and 6 PM, the device beeped on every hour until a mood was logged. In designing this protocol, the disruption of users was minimized while prompting them to think about their emotions.

## Methods

### Ethics Approval

Our study was designed in accordance with our University’s code of ethics and approved by the Open University Human Research Ethics Committee (HREC/3343/Gooch).

### Device

Each of the devices was piloted and was found to induce no discomfort. Participants had the right to refuse to use either of the devices, and it was possible for participants to immediately end their use of a device if they experienced any discomfort. None of the participants opted to do so. We had 2 key concerns in exploring the value of the developed devices. The first is whether participants could accurately record their emotional state through the prototype. The second concern was to explore how our participants used the devices and their view on the design characteristic embodied within the devices.

### Procedure

A field-trial approach was used to evaluate the devices over a period of 6 weeks. This involved each participant taking part in a prestudy session, a midstudy session, and an exit-study session. Each of these sessions took place at a participant’s home and were audio recorded. The sessions lasted between 25 and 54 minutes (mean 28 minutes). Each session was one-to-one between a researcher and participant. Each participant used both devices for 3 weeks. The ordering of which device was used first was counterbalanced between participants as much as possible, although more Emotion Clocks had been manufactured, meaning the majority of participants (n=7) used this device first. The semistructured interview script can be found in Multimedia Appendix 2. The procedure was as follows.

#### Prestudy Session

Sessions began by the researcher explaining that the purpose of the study was to explore new ways of logging emotion and highlighting that no personal emotional experiences would be logged. Informed consent was then collected. Subsequently, this session comprised the following activities: (1) initial data collection, (2) device orientation, (3) emotion logging calibration, and (4) device setup. Each of these activities is described below.

1. Initial data collection: Some basic demographic information was collected from the participant, as well as conducting a short interview regarding any existing logging behaviors (such as keeping a diary), their use of logging technology (such as a Fitbit), and what prompted the participant to take part in the trial.
2. Device orientation: Participants were given a brief explanation of one of the devices (counterbalanced between participants) and how they represent the 2 dimensions of emotion. The researcher answered any questions the participant had regarding the device.
3. Emotion logging calibration: The main element of the prestudy session was to gather data as to whether participants could log emotions using the selected device with the same accuracy as with the standardized paper-based scales. To ensure coverage across different emotional states, standardized emotive vignettes were used. The Affective Norms for English Text (ANET) vignettes are linked to known SAM scores, giving us a known emotion associated with each vignette [66] (referred to as the expected vignette emotion). These texts have previously been used in studies of emotional interfaces [16], as well as with our previous nonfunctional prototypes [31]. For each of the 8 emotions (happy, calm, nervous, excited, sad, relaxed, bored, and annoyed), a short vignette with SAM scores corresponding to that emotion was selected. A condition of using the ANET vignettes is to keep them confidential; so, we are unable to republish them. To illustrate the tone of the vignettes, these 2 examples were written by the first author: (1) “You receive a letter informing you that you have won a holiday to the Caribbean in the quiz you entered last week” (excited) (2) “You discover that your best friend has been diagnosed with a serious illness” (sad). Participants were provided with the vignettes in a randomized order. Having read the text, participants were asked which emotion was portrayed by the vignette. This description is referred to as the participant description. For all of the vignettes, all of the participants provided a synonym of one of the 8 emotions (eg, thrilled becomes excited). The participant description allows us to test that the emotion logged by a participant through the prototype matches the emotion the participant wanted to log. Participants were then asked to record the emotion from the vignette through the prototype. The researcher recorded the result for the prototype alongside the time taken by the participant to record the emotion. Completing this exercise prior to setting the device up means that the logged emotions do not include this initial test.
4. Device setup: The prestudy session ended with the researcher setting the device up within the participants’ home for them to log their emotions for 3 weeks. Participants were instructed that they could place the device wherever they wanted within the home. In terms of use, participants were told that “the device will prompt you to input your emotions twice a day. You can provide more inputs if you wish to.”

At the end of the session, participants were provided with contact details and informed that they could contact us at any time if they were experiencing problems or wanted to talk about the study. We could remotely monitor whether the devices were working correctly by checking the server holding the logged emotions.

#### Midstudy Session

The focus of the midstudy session was to swap over the 2 devices at 3 weeks after the prestudy session. The session started with an audio-recorded wrap-up interview for the device the participant had been using for 3 weeks. The interview covered aspects such as exploring whether the participant had noticed an impact on how they felt, what their general thoughts about the device were, and specific questions regarding the prompting, the aesthetics, the difficulty of interaction, and whether they would hypothetically be willing to share the emotion data they had recorded. Having completed the interview, the researcher swapped over the devices and then repeated the prestudy session with the participant for the second device.

#### Exit-Study Session

Three weeks after the midstudy session, the exit-study session concluded the study and compared the experience of using the 2 devices. The session started with a wrap-up interview for the device the participant had been using for 3 weeks, following the same procedure as for the midstudy session. The session concluded by asking participants to complete a short interview, which was audio recorded. Participants were asked about their general thoughts about the idea of recording their emotions, how hard they found each prototype to use, how hard each prototype was to understand, and their opinions about having a similar device in their home. Further questions explored whether participants continued to be interested in logging how they felt; comparing the 2 devices in terms of use, aesthetics, and how hard they found each prototype to use; and any changes the participant could suggest for improving either of the devices. The study ended with a short debrief, during which time participants were thanked. Participants were shown graphs of their mood data for full disclosure of the collected data. Participants were provided with a £30 (US $39) honorarium for taking part in the study.

### Analysis

In analyzing the data from the study, we had 2 main questions. The first relates to the accuracy of the prototypes: could participants log the emotion they want to log through the prototype devices? The second was to explore our participants’ use of the devices and consider their response to the design characteristics embodied by the devices.

#### Accuracy of the Prototypes

The data from each of the prototypes can be analyzed categorically and ordinally, as outlined previously [31]. As categorical data, there is “ground truth” for each vignette because each vignette is taken from a validated set of emotive texts. Therefore, the emotion the vignette should be provoking in our participants is known (the *expected vignette emotion*). We also have the *participant description*, the emotion the participant believes each vignette expresses. To determine whether the prototypes allow participants to log the emotion they wanted to record, Cohen kappa is used to compare the emotion recorded through the prototype against (1) the *expected vignette emotion* and (2) the *participant description*. Cohen kappa ranges from no agreement (κ=0) to complete agreement (κ=1) [67].

**Figure 5:**
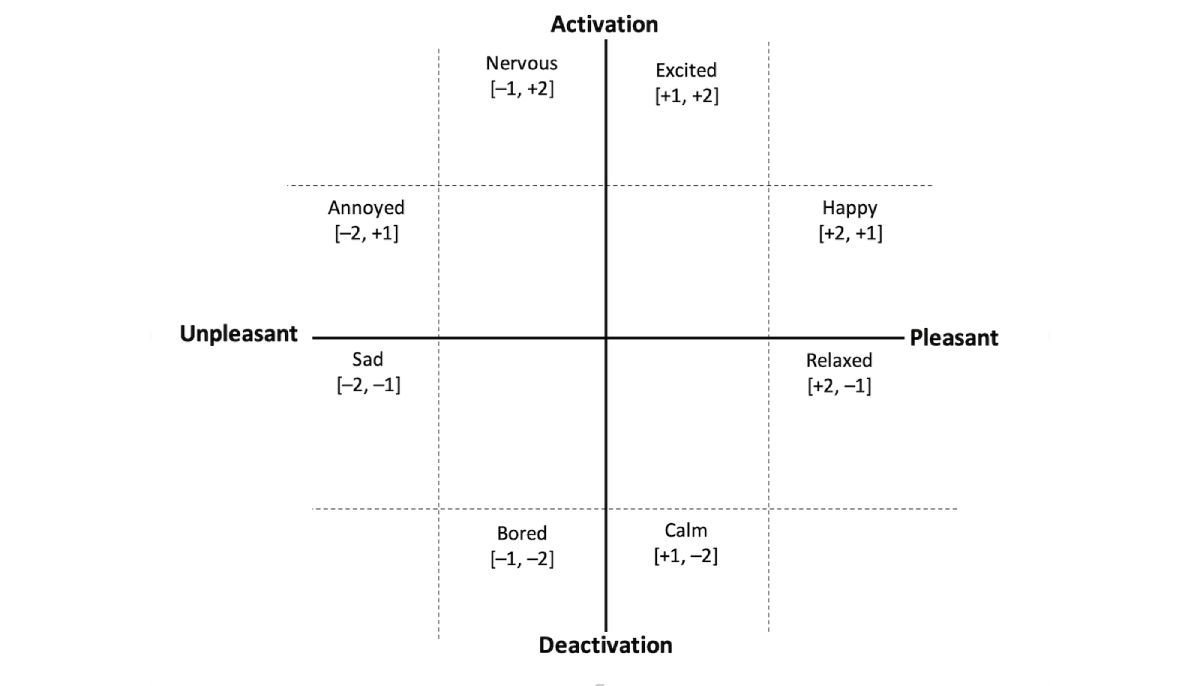
A representation of how the emotions can be given ordinal values on the circumplex of affect.

A problem with treating the data as categorical is that it removes any connection between the different emotions. For example, if a participant records “happy” instead of “excited,” that is a closer match than if they record “sad.” An alternative way of conceptualizing the data is as 2 ordinal scales. Each of the prototypes uses a scale based on Russell’s circumplex of affect (see Figure 2); therefore, each emotion can be represented as a pair of figures ranging from –2 to +2 for both valence and arousal (see Figure 5).

**Figure 6:**
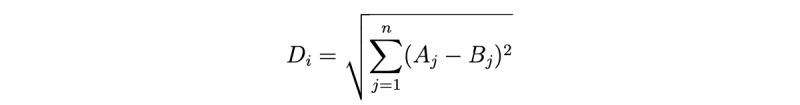
The equation for calculating Euclidean distance.

As an indicator of similarity, it is possible to calculate the Euclidean distance by calculating the distance between 2 matrices (the expected emotional values and the actual emotional values), with each matrix being formed of the valence and arousal values. The distance reflects the size of dissimilarity between the expected emotions and the recorded emotions; the more dissimilar, the greater the distance between them. The Euclidean distance between 2 observations is the length of the line between them. The equation in Figure 6 is used to calculate the distance across all samples. In both the categorical Cohen kappa and the ordinal Euclidean distance, we are not interested in the statistical performance *per se*. Instead, we are looking for confirmation that the prototypes allowed participants to log the emotion they wished to record.

#### Analyzing Participants’ Views

The interviews were audio recorded and transcribed. An inductive open coding approach was used to identify concepts and themes within the interview transcripts [68]. The transcripts were subjected to a line-by-line analysis by the first author, who had not interviewed any of the participants. Through this initial analysis, concepts were identified and labelled within the data. No codes existed prior to the analysis; they were created through constant comparison of the data and the application of labels to the text.

These codes were subsequently categorized into unifying themes by the first author. These themes were there discussed in conjunction with the 3 authors who had interviewed the participants, to ensure that the developed themes corresponded with their interpretation of the data, informed by the interviews they had participated in.

## Results

### Recruitment

Eleven participants were recruited to take part in the study. The study was interrupted by the COVID-19 pandemic. This meant participant #9 could not fully complete the study, as it was not possible to switch the devices over and they only used the Emotion Clock. A further 2 participants (participant #10/ participant #11) could not start the study owing to difficulties of setting the devices up within their homes. Two other participants were somewhat impacted by the pandemic, with participant #5 and participant #8 using their second device, as the United Kingdom went into lockdown. It is unknown whether this had an impact on their logging behavior. We have full data from 8 participants, and partial data from participant #9.

Participants had to be aged over 50 years, be fluent in English, and to have no significant cognitive impairments. Participants’ ages ranged from 51 to 85 years (mean 69 [SD 11.9] years). Seven of our 9 participants were females. All 9 participants had English as their first language. None of the participants reported a history of mental health concerns. Participants were recruited through Age UK Exeter (participant #6, participant #7, participant #9) or personal contact with the authors (participant #1-5, participant #8) through word of mouth or previous participation in other studies. None of the participants had disruptive physical difficulties or cognitive impairments. Table 1 shows the demographics of our participants.

We found no differences in our analysis between those participants who received the Emotion Clock first and those who received the Emotion Board first.

**Table 1:** Demographics of our participants.

Participants	Age (years)	Gender	First device
Participant #1	69	Female	Emotion Clock
Participant #2	74	Female	Emotion Board
Participant #3	69	Female	Emotion Clock
Participant #4	51	Male	Emotion Clock
Participant #5	54	Female	Emotion Clock
Participant #6	85	Female	Emotion Clock
Participant #7	60	Male	Emotion Board
Participant #8	79	Female	Emotion Clock
Participant #9	80	Female	Emotion Clock

### Accuracy of the Logged Emotions

Using standard ANET vignettes provides baseline data of the emotion associated with the vignette, while the *participant description* states what emotion the participant wanted to record. Both can then be compared against the emotions recorded through the 2 prototypes.

The first stage of this comparison is to examine the results as categorical data. Table 2 presents the results from calculating Cohen kappa for each prototype, comparing the emotion recorded in the prototype against (1) the expected result based on the ANET vignette scores and (2) the participant-described emotions. The results show at least moderate agreement (all kappa values>0.5 at *P*<.001) [69], with the Emotion Clock demonstrating strong agreement.

**Table 2:** Cohen kappa values for each prototype.

Prototype	Expected vignette emotion	Participant description emotion
Emotion Clock	0.79	0.91
Emotion Board	0.5	0.5

Examining the results as ordinal data, we calculated the Euclidean distance between the valence/arousal values collected through the prototypes and the expected valence/arousal from the vignettes. The Euclidean distance between the values collected through the prototypes and the participant’s description of the vignette was also calculated. Table 3 shows the Euclidean distances for each of the prototypes. To interpret these figures, it is important to note that there are 64 data points (8 vignettes from 8 participants) on 2 scales running from –2 to +2.

**Table 3:** The Euclidean distance for the valence and arousal data recorded through each interface compared against the expected data from the vignette and the participant description.

Prototype	Vignette total distance	Participant description total distance
Emotion Clock	21.65	18.35
Emotion Board	58.45	21.40

To contextualize the data, we also calculated what the Euclidean distance would be if, for a given interface, all participants were 1 emotion out (see Figure 5, eg, the expected emotion was “excited” and the participant records “happy”). Such a scenario provides a Euclidean distance of 90.51. We also calculated what the Euclidean distance would be if, for a given interface, all participants provided the opposite emotion (eg, the expected emotion was “happy” and the participant records “sad”). Such a scenario provides a Euclidean distance of 286.22. Compared against these contextual calculations, our results in Table 3 show strong-to-moderate agreement between the expected emotion and the recorded emotion. This suggests that the disagreements between expected emotions and recorded emotions noted by the Cohen kappa results were not large discrepancies (eg, logging “happy” instead of “sad”‘) but small (eg, logging “excited” instead of “happy”).

Consistent with the kappa results, these results show a clear difference in the accuracy of the prototype responses with the emotions logged through the Emotion Clock being the closest to both the vignette and *participant description* values.

### Participant Use of the Devices

#### Usage Behaviors

**Figure 7:**
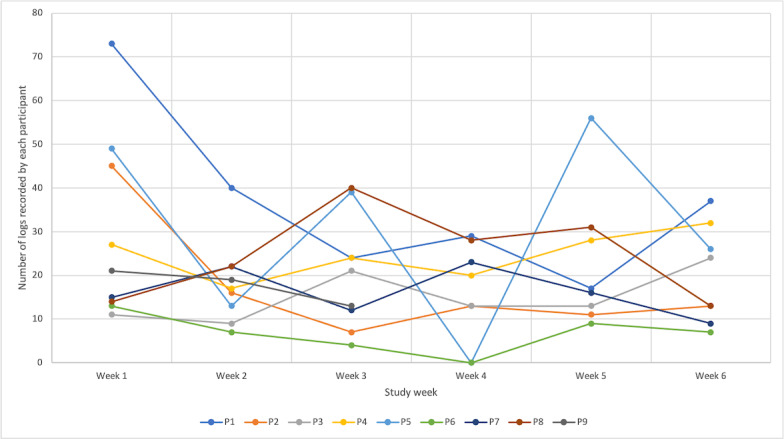
A graph showing the number of logs made by each participant by study week. P: participant.

Having established the accuracy of the devices, we considered the ways in which our participants used the prototypes. Our 9 participants recorded 1085 emotions across the 42-day study (see Table 4). The graph in Figure 7 shows the number of emotions recorded by each participant by study week. This shows some indication of novelty effects (with a high peak for most participants in week 1 and then, a general decline), but the number of emotions recorded is relatively consistent over time.

**Table 4:** Number of emotions logged through the prototypes.

Participants	Emotion Clock (n=579)	Emotion Board (n=506)
Participant #1 (n=220)	134	86
Participant #2 (n=105)	43	62
Participant #3 (n=91)	31	60
Participant #4 (n=148)	63	85
Participant #5 (n=183)	62	121
Participant #6 (n=40)	24	16
Participant #7 (n=97)	93	4
Participant #8 (n=148)	76	72
Participant #9 (n=53)	53	N/A^a^

^a^Not applicable.

In examining the emotions that were logged, there are 3 main groupings, with neutral emotions being logged most frequently (relaxed [n=318], calm [n=276], and bored [n=72]), followed by positive emotions (happy [n=272] and excited [n=76]), with more negative emotions logged rarely (sad [n=31], nervous [n=26], and annoyed [n=14]).

Seven of our participants developed a routine as to when they logged emotions through the devices. Each of these routines was somewhat similar, with all of these participants regularly logging in the morning and evenings, with additional logs throughout the day if seeing the device prompted them to think about logging:

…I have very low energy in the morning. So I usually changed it in the morning. I’d look at it usually, certainly, in the evening as well because at that point I’d be feeling more energetic and lively. During the day, I think, really if... Most of the time, I’m feeling fairly calm and cheerful.

Participant #1

The remaining 2 participants had less of a routine around logging, relying on the prompts from the device or seeing the device as a reminder about logging:

…I don’t think there was any specific time. It was when I suddenly thought, “Oh, I haven’t done that yet,” or I’d been out and I think, “I must do that when I get back.”

Participant #2

All but one of the participants placed the devices in the living room, perhaps the most public area in the home. This decision appeared to be driven by the convenience of using the device in the room most used and where the device would act as a reminder.

For the 8 participants who placed the devices in the living room, there was no concern about their last logged emotion being publicly visible, with their visitors generally being people they would freely discuss their emotions with (friends, family, etc). Instead, the devices acted as a talking point about the purpose of our study, which often led to a discussion of self-reflection:

…I found people were interested in it and often noticed it when they visited, and were interested in the whole idea. I had some friends round, there was quite a long conversation about mood and how you recognize mood. It was a talking point quite a lot of times... when you talked about it, they could recognize that it could be actually quite a clever way of getting you to recognize your mood and to understand how your mood changed.

Participant #1

The participant who did not publicly display the devices, placed them within their study—a room they spend large amounts of time in (and were thus prompted by seeing the device), without advertising their emotions to visitors.

#### Perceived Need to Record Emotion

Five of our participants saw value in the devices as tools to monitor their own emotions, use that monitoring as a prompt for self-reflection and, if necessary, make changes to improve their emotional state:

…it’s a good idea, because it makes you think about your mood, so therefore, you have to think before you select. So where, normally, I wouldn’t bother-I’d just rush through the day.

Participant #5

Eight of our participants also saw the monitoring as potentially a useful mechanism for sharing their feelings over time with other people. This was predominantly in the context of well-being and identifying whether family or friends needed to undertake some action as the person monitoring had seen a persistent or severe change in emotion. Of these 8 participants, 5 would have been happy to share their emotions with loved ones:

…I think I would be more open to indicating than saying probably. That might be just a man thing but it’s you know I mean I just feel that I have to be happy and positive all the time.

Participant #7

The remaining 3 participants indicated that they would be more comfortable with sharing with clinicians (eg, their doctor), would not be comfortable with sharing at all, or could see the value in sharing but did not feel they were at that life stage yet (which did not correspond with participant age). This led us to consider whether participants who were less willing to share had a different profile of logged emotions (eg, whether they had a greater percentage of negative emotions). Comparing the participants’ willingness to share their logged emotions with the emotions that participants had logged through the devices did not establish a clear pattern, with willingness to share more likely related to an individual’s feelings of privacy.

#### Use of the Devices

Having noted that most participants identified a perceived need for the devices, it is necessary to consider what evidence there is that the devices had value to our participants. Five of our participants found that both of the devices helped them reflect on their emotions, with another 2 participants reporting this was only the case for the Emotion Clock and the Emotion Board. The ability to regularly log an emotion was a sufficient prompt to provide a scaffold for all of these participants to reflect on their emotional state:

…I think I thought about my moods quite a bit more, how I was really feeling, you know... Using it has had a positive impact, yes, because I’ve had to really think about how I feel.

Participant #2

This was particularly the case during significant occasions. For participant #1 over their birthday and for participant #2 when their dog died, they found that the devices were particularly helpful in encouraging them to reflect on how they were feeling.

Most of our participants would like to continue using the devices. When explicitly asked whether they would like to continue monitoring their emotions using our devices, 5 of the participants saw clear value in them and would like to continue using them. None of these participants expressed a preference for only continuing with one of the devices. The remaining 4 participants did not like to continue using the devices, mainly as they did not perceive any derived benefit from their use. This included the 3 participants who did not consider themselves at a life stage of needing such a device; therefore, their disinterest was not a matter of dislike but rather of current lack in perceived need for emotional well-being management.

#### Device Preferences

Although the devices share certain design characteristics, the nature of interaction is significantly different. The clock offers a quick, immediate, and limited choice, while the board offers a more open-ended exploratory wide-ranging selection. It is worth examining how our participants engaged with these distinct designs and what can be learnt from those engagements.

For the Emotion Clock, 7 participants praised the simplicity of the design, stating:

“it was easy enough to use.”

Participant #9

These participants went on to discuss how the specificity of the emotions listed was not necessarily the emotions they wanted to record:

…I am, actually, a very busy person, which is why I say you should have that on there. If you’re busy, you’re not necessarily relaxed or calm. (Laughter) You’re just busy. Obviously, ‘lonely’ is not on here.

Participant #3

This raises a question of the value of customizability, but in personalizing the words available for participants to select, the link between the device and the underlying psychological scales is removed. In contrast, only 2 participants felt that the Emotion Board (participant #3 and participant #4) was easy to use. Four participants felt that the Emotion Board was relatively difficult to understand, with the open nature of the interaction causing confusion:

…I sometimes found it a bit difficult to quite understand the square. I tended to move the thing round the edges of the square, I wasn’t sure how the middle works and whether that calibrated things differently into the center.

Participant #1

For some participants, this meant that they did not feel comfortable exploring the range of options through the Emotion Board, thereby reducing the use of the device as they did not understand the continuum nature of the design. However, 5 participants felt that while the Emotion Board was harder to understand, the necessary thought could help provoke further engagement and reflection:

…I had to think about that more... I certainly had to think about it more than with the [Emotion Clock], because it was whether you were feeling up, down, you know, energized, not energized.

Participant #3

Participant #4 also noted that they related more to associating feelings with colors than they did with words, making the Emotion Board much more meaningful for them.

When our participants were asked which of the devices they preferred, the Emotion Clock was the most popular choice, with 6 of the participants preferring the simplicity of the interaction and the visual design. The other 2 participants, participant #4 and participant #8, preferred the open-ended interaction of the Emotion Board.

### Design Characteristics

Having explored the specific design qualities of the individual devices, it is worth considering the design characteristics the devices shared and how they influenced our participants. The 2 devices shared certain design characteristics, particularly a shared aesthetics and a shared prompting system.

Six of our participants discussed the aesthetics of the devices without being prompted. All 6 were positive about the designs, noting that constructing the devices from wood made the devices pleasant to look at and made them blend in to the home environment. This is important as the aesthetics of the devices are likely an important factor as to whether people are likely to use the devices for long-term use; we would argue that if people are pleased by having the device in the house, they are much more likely to engage with the emotion logging in the long term.

As reported earlier, only 2 of our participants relied on the prompts for logging emotions, with the other 7 participants developing their own routine. All of the participants noted that the audio prompting was not annoying and not distracting. Participant #1 noted that on occasion, the prompt could be useful as an occasional reminder, while participant #2 suggested increasing the frequency to 4 times a day as a more regular prompt. In general, though, our moderate prompting appears to have been appropriate.

## Discussion

### Value of the Devices

The focus of this work has been in evaluating the value of our tangible emotion logging devices for older adults. Our results demonstrate that our tangible devices can record data comparable to psychological scales of emotion. Such a finding validates the use of TUIs in this context and demonstrates that such devices could hold value for older adults. Furthermore, the level of use of the devices from our participants indicates that the participants saw some value in using the devices. The devices hold certain design properties that supported this use, particularly reflection on sparse data, provision of no data history, and focus on minimal interactions.

These properties are not unique in research into reflective logging technology. The value of reflecting on sparse data with minimal history is attracting increasing attention [70,71]. Further, focusing on minimal interaction is seen as a way for users to log meaningful data without becoming overburdened by the effort of logging [63,64,72]. We have built on this work and demonstrated that these design qualities in a different context—tangible devices for older adults—can support meaningful emotional reflection. Our findings open the design space for further consideration of how tangible devices can support emotional logging and reflection.

More specifically, our work also contributes to 2 ongoing interrelated debates within the field: the role of reflection in designs such as ours and the value of customizability in logging devices.

### The Role of Reflection

Along with much of the human-computer interaction field, we have been somewhat imprecise in our treatment of reflection in our work, providing no firm definition or placing it within a theoretical framework [73]. To a certain extent, this was deliberate—our interest has been more on the design and success of the device rather than the mechanism through which users gained value. Although we operate under the assumption that the act of logging an emotional state would prompt users to think about their emotions and more broadly, their well-being in a form of reflection-in-action [58], we have not attempted to demonstrate that this mechanism is how our users gained value from the devices.

One of the key debates over supporting reflection through interaction design is the process by which reflection occurs. The model from Li et al [74] argues that reflection only happens at 1 stage of the reflection life-cycle, after preparation, collection, and integration, with the reflection leading to an action. This contrasts with the model from Epstein et al [61], which is more cyclical, with reflection taking place during an activity as well as afterwards.

Our research supports work that has demonstrated that people can reflect on relatively sparse data [75]. Our results suggest that a simple interaction, with no recorded history, is sufficient to support some users in reflecting on their emotional state. This is much closer to the Epstein et al’s [61] model of reflection. None of our participants requested to see their recorded data at any point during the study, further suggesting that focusing on the design of the logging experience rather than on the historical record could be more beneficial to users.

One of the aims of personal informatics is to support behavior change and self-improvement by helping people become more self-aware. Some researchers have proposed that to do this effectively, we should not be constrained by supporting the consideration of past events but provide recommendations for future actions [76]. Such systems involve a combination of different subsystems. These include interfaces and device development, the design of analysis algorithms, and a complex sociotechnical mechanism for supporting the recommended actions.

Instead of attempting to construct all of the elements of such a system, we have focused on a single element (the interface design and device development), with results indicating that well-designed interfaces can be sufficient for some people to derive value from them. It remains an open question for the field as to whether such results can be enhanced by connecting such an interface to a well-designed and validated sociotechnical system for supporting deeper reflective actions. Given the complexity of the necessary “ongoing negotiation of the boundaries and meanings of self within an anxious alliance of knowledge, bodies, devices, and data” that is necessary for effective long-term use of logging technologies [77], we have provided a starting point for exploring the value of tangibles in this alliance.

### The Value of Customizability

Some participants noted that they would have liked to have been able to customize the devices so that they were logging emotions more linked to their day-to-day experiences. Although this is perfectly feasible from a design perspective, it does remove the link between the device design and the underlying validated psychological scales being used. Our focus on ensuring the devices are linked to the validated psychological scales comes from the broader context of this work, where the research team is part of a project investigating home-based health monitoring technology. Working with clinicians, there was a focus on ensuring that if the data were later to be shared with clinicians or other stakeholders, it would be possible to understand the data in the context of an established framework.

This dichotomy is representative of a long-standing concern within the personal informatics community, with some researchers exploring better ways of aggregating and analyzing precise quantifiable data [78,79] and others arguing for a switch from a focus on “behavior and its objective data to the self and its subjective meanings” [71].

An alternative approach would be to design around affect labelling. This regulation technique can be described as asking people to put their feelings into words [80], which can help people regulate their emotions [81]. This could prove an interesting route of customization for 2 reasons. First, it would be aggregating the labels in a meaningful way so that the historical record is useful to both the person logging and any related need (eg, with a clinician or carer). If the labels were restricted to a wide (but standardized) set such as Plutchik’s Wheel of Emotions [82] or the Geneva Emotion Wheel [83], this aggregation could still take place automatically. Second, given the value of affect labelling comes from its open-ended nature, this is a design challenge in translating such a technique into a tangible logging tool.

### Limitations and Further Work

We are working in an imprecise area of human experience. This means our findings and conclusions must be tempered by known limitations as discussed below.

Our first limitation stems from the design decisions we made. First, the Emotion Board makes strong use of color. Color is an inappropriate prompt for people with color blindness, and we have not accounted for the cultural implications inherent in color. Second, our devices do not cover fleeting emotions, as discussed by 2 of our participants. Third, by focusing on tangible technology suitable for the home, the resulting design was not suitable for logging emotions in outside contexts, as noted by 3 participants. Although we acknowledge these limitations as properties of our designs, they also indicate promising directions for further work.

The study methodology has a limitation in that we are unable to report the extent to which the participants’ accuracy of interpreting the emotion expressed in the ANET vignettes was influenced by their personal ability to understand other people’s emotions or their personal emotional reactions to the stimuli. We decided against screening participants based on their ability to interpret emotions from the vignettes and compensated for this by asking for the *participant descriptions*.

Additionally, we have no mechanism for comparing the data that participants logged during the field trial and how those participants were actually feeling. Although none of the participants raised this as an issue during the interviews, we cannot be completely certain as to whether participants tended to underlog or overlog particular types of emotions. Methodologically, this remains a challenge.

More broadly, our participant pool is relatively small and further work is needed to explore the generalizability of our results. The size of our study was directly limited by the COVID-19 pandemic, with one study cut short (participant #9) and 2 recruited participants unable to take part (participant #10, participant #11). Given that we were unable to safely distribute the tangible artefacts to a particularly COVID-vulnerable population, we were unable to extend the number of participants within the study. Furthermore, 6 of our participants were recruited through contact with the authors through word of mouth or previous participation in other studies. Although we have no personal relationship with these participants, they are more likely to be engaged in this kind of research and more technically able than the population as a whole. This convenience sampling also led to a gender imbalance among our participants. While limiting the strength of the evidence, we are not arguing that our results are replicable across the population at large, but we argue that our work provides promising results and indicates further research directions.

### Conclusion and Future Work

In this paper, we have contributed one of the first empirical investigations into the suitability of using tangible devices based on standardized scales of emotion for older adults to log emotions. We conclude that our devices are sufficiently accurate in collecting emotional data from older adults. Additionally, our work demonstrates the potential for using tangible devices to assist older adults in logging their emotional state to support reflection and emotional well-being. We argue that there is a significant amount of future work needed to extend this work by exploring whether this value holds when using tangibility as a design property of more self-expressive logging technology for older adults. Given the sharp divide between the competing interests of generalizability and customizability, it is clear that designers have to establish what is more important to their user base. They should also ensure that their users have alternative options if their preferences change over time.

We argue that this success highlights the suitability for tangible devices to be used for long-term logging within the home. This study provides foundational support for tangible emotion self-logging devices for older adults and justifies further large-scale field studies exploring the effects of each device type on long-term engagement. In future work, we plan on exploring 2 interrelated aspects: (1) whether tangibility can be developed as a design quality for more self-expressive logging technologies and (2) exploring how to develop resilient sociotechnical support that responds to the data being logged by older adults. In doing so, we will better understand how tangible devices can help older adults wanting to maintain and improve their long-term well-being.

## Appendix

Multimedia Appendix 1 Emotional logging summary tables.

Multimedia Appendix 2 Interview questions.

## Abbreviations

ANET: Affective Norms for English Text
SAM: self-assessment manikin
TUI: tangible user interface
