# Supplementary material for: Designing Tangibles to Support Emotion Logging for Older Adults: Development and Usability Study
Source: JMIR Hum Factors. 2022 Apr 27;9(2):e34606. doi: 10.2196/34606 (PMC9096637; doi:10.2196/34606)
Supplement: Multimedia Appendix 1 [file humanfactors_v9i2e34606_app1.pdf]

Multimedia Appendix 1: Emotional Logging Summary Tables

Reference	Study Focus	Study Approach	Key Outcomes or Design Criteria
Zhang et al, 2019 [49]	Mental Health Apps	8-week randomized trial of 13 apps	Self-tracking led to reduction in depressive symptoms. Minimal engagement sufficient to support users.
Goodday et al, 2020 [45]	Mental Health Apps	Used widely across the UK	Advantages: prompting, visualization over time
Chandrashekar, 2018 [50]	Mental Health Apps	Meta-study review	Simple user interfaces Minimal usage reminders
Fernández et al, 2017 [52]	Older adults	Digital Diary	Minimal logging sufficient to support users
Broekens et al, 2013 [16]	Emotion logging	Range of large scale studies	Possible to use digital interfaces to collect meaningful emotive results
Rivera-Pelayo et al, 2017 [20]	Emotion logging	Field study	Digital interface can support team communication
Morris et al, 2010 [51]	Emotion logging	Mobile app field trial	Mood logging can lead to meaningful personal change
Rodríguez et al, 2015 [53]	Tangible	Device development, focus group	Suitable for participants with low digital competence; importance of form factor
Balaam et al, 2010 [54]	Tangible	Field-trial in schools	Supported emotional reflection; importance of form factor
Sarzotti , 2018 [17]	Tangible	Discussion session	Interest in the tangible form factor; importance of form factor
Jingar et al, 2019 [55]	Tangible	Co-design with older adults	Tangible interfaces suitable for use by older adults; importance of form factor
Gooch et al, 2020 [31]	Tangible	Emotion logging paper prototypes	Tangible interfaces suitable for older adults to log emotion; importance of form factor
