# Supplementary material for: Designing Tangibles to Support Emotion Logging for Older Adults: Development and Usability Study
Source: JMIR Hum Factors. 2022 Apr 27;9(2):e34606. doi: 10.2196/34606 (PMC9096637; doi:10.2196/34606)
Supplement: Multimedia Appendix 2 [file humanfactors_v9i2e34606_app2.pdf]

Version 3.0 (4/11/2022)

# Mood Self-logging Prestudy Interview Script

**Participant ID** ______________ **First Device** ____________

1. Previous/current profession

2. Have you ever kept a diary of any kind? Do you now? If so, do you write about your moods?

3. Do you have/use a fitbit or other similar device?

4. What prompted you to take part in this trial? What do you hope to get out of it, if anything?

---

- Run through how the device works.
- Refer to 8 vignette texts. Provide them in pre-randomized order and ask the participant to express corresponding mood on the device for each vignette.
- Record any difficulties the participant had in using the device – both in understanding the device and motor skills

# Mood Self-logging Post Device Use Interview Script

**Participant ID** ___________ **Device/Deploy order** ___________

1. Administer WHO-5 Wellbeing Index

2. Having used the Mood [device] for 3 weeks, what do you think about the idea of logging your mood?

3. As a result of using the device, did you reflect more or less often on how you felt? Did it have a positive or negative impact on your mood?

4. When did you typically record your mood through the [device]?
   a. When prompted?
   b. Times of day/visitors/salient events?

5. How did you find the prompting? Useful, annoying, too frequent, didn’t notice?

6. What were your general thoughts about the [device]?

7. How easy or difficult was it to use?

8. Could you log the moods you wanted to? Were there moods you wanted to log that you couldn’t? If so, which?

9. Are there moods available that you wouldn’t use? And if so, why?

10. What did you think about how the device looked?
    a. where in the house did you put it and why?
    b. were you happy for other people to see it and why?
    c. did other people comment on it? What did they say?
    d. Did the location of the device influence what you chose to log?

11. Are there any changes you would suggest to improve the [device]? What and why?

12. How do you feel about others having access to your current mood? How do you feel about sharing your mood history with others? Who would you share with? Who would you not share with? In what circumstances/situations?

13. If this was first device, demonstrate second device and record any difficulties the participant had in using the device – both in understanding the device and motor skills.

# Comparison Self-logging Final Interview Script

## Participant ID ______________

1. Having logged your mood for 6 weeks, would you continue doing so in any way? Why?
   a. Would you continue using either of the devices? If so, which one and why?

2. Having used both devices, which did you prefer? Why?

3. Which did you find easiest to use? Why?

4. Which did you find easiest to understand? Why?

5. What would you think about having either of these devices in your home? Which ones and why/why not? Which rooms would you be comfortable having them in?

6. Are there any changes you would suggest to improving either of the devices? What and why?

7. Do you have any other comments or suggestions?

8. Show visualizations and get comments
